# Statistical Platform for Individualized Behavioral Analyses Using Biophysical Micro-Movement Spikes

**DOI:** 10.3390/s18041025

**Published:** 2018-03-29

**Authors:** Elizabeth B. Torres, Joe Vero, Richa Rai

**Affiliations:** 1 Psychology Department, Rutgers University, Piscataway, NJ 08854, USA; richarai9@gmail.com; 2 Computer Science Department, Computational Biomedicine Imaging and Modeling, Rutgers Center for Cognitive Science, Rutgers University, Piscataway, NJ 08854, USA; 3 Bioengineering Department, Rutgers University, Piscataway, NJ 08854, USA; joev714@gmail.com

**Keywords:** micro-movements, spikes, peaks, stochastic analyses, *Gamma* process, activity tracking, activity classification, motor control

## Abstract

Wearable biosensors, such as those embedded in smart phones, can provide data to assess neuro-motor control in mobile settings, at homes, schools, workplaces and clinics. However, because most machine learning algorithms currently used to analyze such data require several steps that depend on human heuristics, the analyses become computationally expensive and rather subjective. Further, there is no standardized scale or set of tasks amenable to take advantage of such technology in ways that permit broad dissemination and reproducibility of results. Indeed, there is a critical need for fully objective automated analytical methods that easily handle the deluge of data these sensors output, while providing standardized scales amenable to apply across large sections of the population, to help promote personalized-mobile medicine. Here we use an open-access data set from Kaggle.com to illustrate the use of a new statistical platform and standardized data types applied to smart phone accelerometer and gyroscope data from 30 participants, performing six different activities. We report full distinction without confusion of the activities from the Kaggle set using a single parameter (linear acceleration or angular speed). We further extend the use of our platform to characterize data from commercially available smart shoes, using gait patterns within a set of experiments that probe nervous systems functioning and levels of motor control.

## 1. Introduction

We are entering an era of Precision Medicine [1] and mobile health, where personalized assessments at the clinic are combined with follow up assessments on the go. In the areas of mental health, this new platform is bound to revolutionize basic research and clinical practices [2], particularly if current subjective behavioral evaluations begin to be complemented with more objective and automated behavioral analyses [3]. Along those lines, wearable sensing technologies are destined to help accelerate the transformation of medical and scientific practices, particularly in the areas of mental health.

Wearable sensors listening to the activities that are self-generated by the person’s nervous systems can help harness such biorhythmic activities in non-invasive ways. In this sense, such “biosensors” have become ubiquitous in our lives and can be found embedded in smart devices and activity trackers that we commonly carry with us. The time series of signals they output can help us self-monitor the various levels of functionality of our nervous systems. When paired with proper analytics, these biosensors’ activity can help us ascertain levels of volition and autonomy of the brain over the body in motion [4].

Yet how do we take full advantage of such time series data and produce verifiable and reproducible results that maximize objectivity and automate statistical inference?

**Figure 1:**
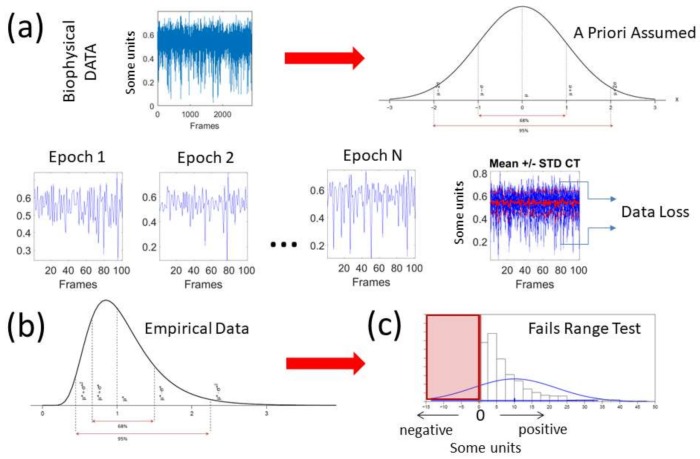
Incorrect statistical assumptions about biophysical data. (**a**) Time series of biophysical data are often analyzed using theoretical assumptions of normality that do not correspond with actual data. Conventionally, epochs of data are stacked up and a Gaussian mean is used to summarize the data range and its variations, thus smoothing out as noise relevant fluctuations and incurring in gross data loss; (**b**) Very often, the empirical distributions and/or their fluctuations are not symmetric. (**c**) The empirical data may fail the range test, while parametric statistical tests and further inferences are based on assumptions of normality (i.e., symmetric distributions).

The deluge of data throughput that biosensors produce when continuously monitoring activities of the nervous systems over months and years can be challenging to analyze, interpret and decide upon. Part of the problem is the “one size fits all model” approach to the analyses (Figure 1a). Traditionally, several assumptions are made about the data that are not empirically informed. Often, common biophysical signals (e.g., from the amplitudes of speed, acceleration, heart rate, electroencephalography (EEG), electromyography (EMG)) reveal peaks and valleys that convey information about fluctuations in the activity of the nervous systems. Under the Gaussian theoretical assumptions, these fluctuations are smoothed out through grand averages and treated as noise. Consequently, the very information we are seeking to read out from the nervous systems can be lost. Statistical inferences from such theoretical treatment of the biophysical data are incongruent with the skewness found in empirical data [5] (Figure 1b). The data of interest may even fail the range test under such pre-imposed (symmetric) theoretical assumptions (Figure 1c). One may end up inferring and interpreting data that falls out of the actual range of values of the empirical data. For instance, consider speed peaks frequently used as a feature of a parameter of interest. Under some experimental task, the linear speed under consideration may have amplitudes ranging from near zero (no movement) to some value, say 50 cm/s. Under repetitions of the task, the researcher finds that the assumed theoretical Gaussian mean (µ) yields 10 cm/s and the standard deviation from the mean (σ) comes out high to 7 cm/s (perhaps this task requires adaptation and gives rise to high variability in its initial stages). When one takes μ ± 2σ, there are (assumed) variations in the non-existent negative range of that empirical data (i.e., negative speed does not make physical sense). The researcher may go on to make many inferences and further interpretations of the data under assumptions that were not empirically justified. This hypothetical situation is in fact very common in the scientific arena.

Another problem preventing us from making full use of the richness of biophysical data is the lack of a standardized metric scale. Such a scale would permit proper comparison across heterogeneous populations suffering from a common nervous system disorder. For example, motion parameters such as linear and angular speed depend on the anatomical dimensions of the person (because speed is distance traveled per unit time). Measurements of speed taken at a fixed unit time will be affected by anatomical proportions (bone length). If a study participant has longer limbs than another participant, the speed amplitude values will be higher and the range of values may also be affected by such allometric issues. Neurodevelopmental data is particularly sensitive to this problem, as physical growth occurs within days at early neonatal stages. The underlying peripheral nerves, carrying efferent and afferent flows of information, change at irregular and accelerated rates [6]. During early stages of neurodevelopment speed-dependent data is particularly susceptible to non-uniform and non-linear changes in the stochastic signatures capturing the inherent variability of the data. The shape and dispersion of the probability distributions empirically characterizing such data shift rather fast and irregularly. Yet, often the data are examined using a static, linear framework under assumptions of normality. Such data will have to undergo some form of standardization [3,7] to allow for comparisons across different ages (e.g., young babies vs. four- to five-year-old children). 

Nervous systems with different pathologies adapt and cope with the problems as the person develops and ages. This biological process takes place at rates of change that are unique to each person. How is the nervous system of a person coping with a disorder changing in relation to that of other people with a similar diagnosis? Diagnoses are based on expert clinical opinion derived from observation. As such, the symptom-based classification results in highly heterogeneous phenotypes [8]. Given a clinical intervention then, what does it mean to converge towards a normal level? Once again, the current methods that rely on grand averages and a one size fits all model cannot address such complex non-linear dynamically-changing phenomena. Whether building normative data sets or analyzing data from individuals with complex pathologies of the nervous system, it will be critical to empirically inform our methods, particularly those relying on machine learning (ML) algorithms, so commonly used to examine time series of biophysical data from wearable sensing devices.

The medical field has shifted gears towards a paradigm that complements clinical expertise from humans with methods used in artificial intelligence (AI). The latter is aimed at compiling large amounts of data and efficiently integrating information from multiple layers of the knowledge network, ranging from patient self-reports to clinical scores from subjective behavioral observations to genomic data. Often the goals are to aid the process of decision-making during treatment selection and patient care that may potentially lead to better quality of life. Along those lines, the combination of open access big-data sets with ML techniques has been instrumental in parameter identification and identification of features that maximize information inherently present in the time series data, with the potential to detect patterns of relevance to a disease or its progression.

One active site that has contributed tremendously to this mission of uncovering analytics to make biosensing data analyses automated and objective is Kaggle.com. This site maintains several repositories of human activity data and makes all aspects of the data acquisition specs available to the public. Such initiatives permit the development and testing of new methods, along with their dissemination for reproducibility and improvement across the research and clinical communities.

In this paper we employ one such data set to re-analyze it, using a heuristics-free objective method that (1) provides a new standardized data type amenable to build proper statistical scales for personalized and population analyses; (2) automatically uncovers clusters of activity types; (3) provides methods to build maps of general population features.

The first part of the paper describes the results of applying our methods to the Kaggle.com activity set [9] as an alternative to other approaches (e.g., [10,11,12], etc.) that use ML methods. The second part of the paper then illustrates the methods using commercially available wearable sensors to underscore the empirically-informed statistical inferential properties of our statistical platform that remain invariant to sensor type, data parameter, or features extracted from the data. We highlight the utility of the methods to produce a standardized scale amenable to profile the general population. We also provide (upon request [4]) libraries in Matlab (The MathWorks, Inc., Natick, MA, USA) and Python Programming Language (Wilmington, DE, USA) for use by the general community and aimed at promoting reproducibility of results.

## 2. Materials and Methods

The methods described in this paper have been previously published. See illustrative examples of the use of this platform in Appendix A. For simplicity, we summarize them here in two steps: (1) the definition of the standardized data type and (2) the description of the analytics. The data sets used to illustrate the methods are from (1) Kaggle.com (https://www.kaggle.com/uciml/human-activity-recognition-with-smartphones) containing the set of “Human Activity Recognition with Smartphones”. (2) Our Sensory Motor Integration Lab (SMIL) at Rutgers University (Piscataway, NJ, USA). The human data was collected under the protocol approved by the Institutional Review Board of Rutgers University in compliance with the Helsinki Act.

### 2.1. Data from Kaggle.com

**Figure 2:**
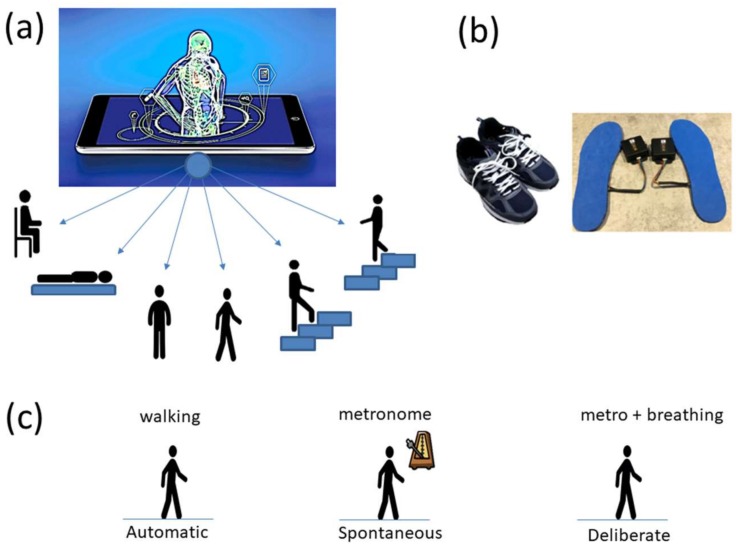
Experimental conditions. (**a**) The Kaggle activity recognition set includes 6 activities recorded using accelerometers and gyroscopes embedded in a smart phone (sitting, laying, standing, walking, walking upstairs and walking downstairs). (**b**) The Sensory Motor Integration Lab (SMIL) data set includes gait patterns collected with Zeblok smart shoes containing inertial measurement units and pressure sensors employed to measure natural walking. (**c**) Further variations on natural walking are used to assess sensitivity of the insole sensors and to profile levels of neuro-motor control across automatic, spontaneous and deliberate states.

The following description of experiments are taken from the Kaggle website and are illustrated in Figure 2a. We quote:

- “The experiments have been carried out with a group of 30 volunteers within an age bracket of 19–48 years. Each person performed six activities (WALKING, WALKING_UPSTAIRS, WALKING_DOWNSTAIRS, SITTING, STANDING, LAYING) wearing a smartphone (Samsung Galaxy S II) on the waist. Using its embedded accelerometer and gyroscope, we captured 3-axial linear acceleration and 3-axial angular velocity at a constant rate of 50 Hz. The experiments have been video-recorded to label the data manually. The obtained dataset has been randomly partitioned into two sets, where 70% of the volunteers was selected for generating the training data and 30% the test data.
- The sensor signals (accelerometer and gyroscope) were pre-processed by applying noise filters and then sampled in fixed-width sliding windows of 2.56 s and 50% overlap (128 readings/window). The sensor acceleration signal, which has gravitational and body motion components, was separated using a Butterworth low-pass filter into body acceleration and gravity. The gravitational force is assumed to have only low frequency components, therefore a filter with 0.3 Hz cutoff frequency was used. From each window, a vector of features was obtained by calculating variables from the time and frequency domain.”

We analyze the data from all 30 participants (accelerometer and gyroscope) and also the summary data (mean acceleration and angular speed). We take the peaks detected by the accelerometers at the corresponding sampling rate. For example, at 50 Hz, 30 s worth of data provide 1500 frames with well over 100 peaks necessary for robust estimation. In this sense, our criteria for number of peaks in a segment is that they yield tight confidence intervals for the estimated parameters generating the probability density function (PDF) curve to fit the frequency histogram. Our methods do not require the training/testing partition that ML methods employ to analyze these data. We mixed all data from all participants into a full data set. Further, we only use the acceleration and angular velocity data with a focus on fluctuations of peak amplitude and peak timing. We do not use other features in the Kaggle.com set including frequency domain parameters or information theoretical parameters.

### 2.2. Data from Rutgers SMIL

Commercial Sensors (Zeblok, Ridgefield, NJ, USA, smart shoes): One data set from one participant consists of 6 activities (WALKING, JUMPING, STEPPING AROUND OBSTACLE, STEPPING OVER OBSTACLE, WALKING ON A TREADMILL and RUNNING ON A TREADMILL.) Another data set from the same participant consists of three variants of the walking task. The participant in these sets wore Zeblok smart shoes which are commercially available. The shoes have IMU (inertial measurement unit) that collect data at 150 Hz (specs can be found here http://www.zeblok.com/). Figure 2b shows the insoles the participant wore inside regular tennis shoes (removing the original insole and replacing it with the Zeblok one.) Figure 2c shows the schematics of another set of tasks previously described [13], designed to probe the levels of control of the nervous systems: (1) natural walking (baseline) to probe ‘automatic control’; (2) metronome condition whereby a metronome is set in the background to probe ‘spontaneous control’, i.e., test if the biorhythms of the person spontaneously entrain with the metronome’s rhythm and to test if the brain is capable of spontaneously adapting to the new tempo; (3) breathing condition where the person is instructed to deliberately breathe to the metronome’s beat and we test the level of ‘volitional control’ of the person over their gait motions.

Note here that the use of one participant and set of tasks to test these sensors was not meant to compare the outcome with that of the Kaggle.com set. These are independent tasks registered with different sensor types (Kaggle set sampled at 50 Hz whereas Zeblok sampled at 150 Hz). The purpose of adding the one subject tasks was to test if the analytics could extract differences in fluctuations even when the tasks involved subtle variations of a basic activity such as walking.

Walking was present in all sets of tasks and sensor types. In the Kaggle.com set, there are two main families of tasks. One requiring motor control during overt motions of variable dynamics: walking, walking upstairs and walking downstairs. In contrast, the other set required covert motions: sitting, standing and laying down. Such tasks require motor control to keep the body in place. The natural walking task common to both sets (Kaggle.com and SMIL-collected with Zeblok insoles) posed the main goal to test if the analytics could differentiate the cases where subtle variations in breathing and external audible rhythms were present. In the Kaggle.com it was somewhat obvious that the overt vs. covert motion tasks differed. As such, it would not be as surprising if the analytics could distinguish variations in each activity class. Would these analytics be able to distinguish subtle variations within the natural walking task? Metronome induced variations and variations induced by deliberately breathing to the rhythms of the metronome are rather subtle. It was not obvious that these analytics would distinguish among such variations of the natural walking task.

### 2.3. Spike Trains Extracted from Behavioral Data (the Micro-Movements)

The time series data of triaxial acceleration and gyroscope vectors were converted to scalar time series data using the Euclidean norm to obtain the magnitude of each vector in the time series. This produced a time series of peaks and valleys characterizing the acceleration and angular speed. Two features of interest in each time series were the fluctuations in peak amplitude and the fluctuations in the times these peaks occurred. The peaks (and times of the peaks) were gathered into a frequency histogram to estimate the overall mean of the data set for each activity. To that end, we estimated the best continuous family of probability distributions fitting the data (using maximum likelihood estimation (MLE)). This turned out to be the continuous *Gamma* family of probability distributions (confirming prior work on movement-based biometrics [4,5]).

The *Gamma* distribution has two parameters, the shape (*a*) describing the shape of the distribution (e.g., symmetric, skewed, etc.) and the scale (*b*) describing the dispersion. We estimate each of these parameters with 95% confidence intervals using MLE.

A random variable *X* that is *Gamma* distributed with shape *a* and scale *b* is denoted by X∼Γ(a,b)≡Gamma(a,b) with probability density function:

(1) f(x;a,b)=xa−1e−xbbaΓ(a) for x>0;a,b>0.

The *Gamma* mean μ=a·b and the variance $\sigma =a\cdot b^{2}$ with the noise to signal ratio $NSR=\frac{\sigma }{\mu }=b$, the scale parameter that we will refer to as the noise.

**Figure 3:**
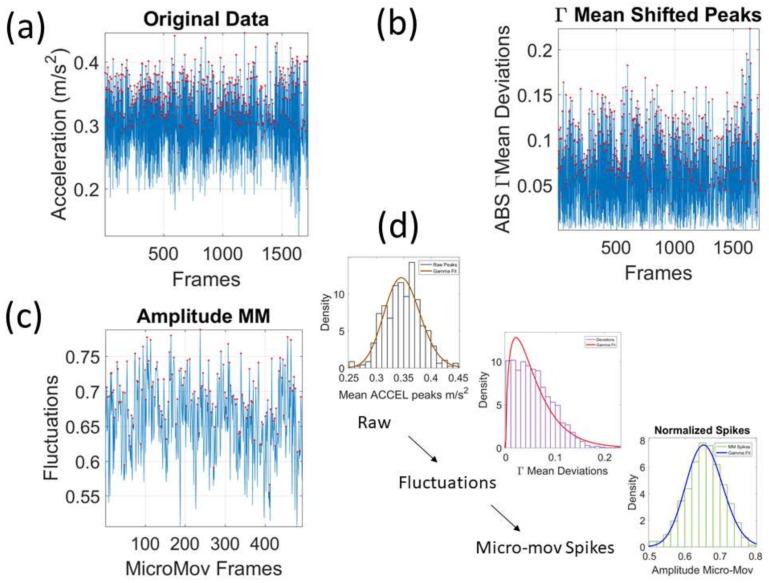
Pipeline of data analyses to construct the micro-movement spike trains. (**a**) Raw acceleration data with peaks marked by red dots (each peak is detected by a change in the slope from positive to negative trend). (**b**) Overall mean is empirically estimated and the absolute deviations are obtained and plotted for each frame. (**c**) The peaks are extracted and normalized using Equation (2). (**d**) Distributional analyses are performed to determine the best continuous family of distributions at each step fitting the frequency histograms of fluctuations in peak amplitude or peak timing.

We mean-shift the original data (shown in Figure 3a, e.g., for walking condition) to center it at the mean value and obtain the absolute deviations from the estimated *Gamma* mean (Figure 3b). Then, for each local peak fluctuation in amplitude (or timing) away from the original series estimated-*Gamma* mean across all frames of the acceleration (or angular speed) profile of each routine segment, we scale it and produce a normalized value of the peak:

(2) $$Normalized\;Peak=\frac{Local\;Peak}{Local\;Peak+Local\;{Average}_{min-to-min}},$$

where the peaks and the local average values computed between the local minima surrounding the peaks are *Gamma* distributed. The frequency histogram of the average acceleration (m/s^2^) (or angular speed rad/s) produces a unitless quantity, which we coined the micro-movement spikes. The unitless micro-movements (MM) representing the real-numbered spike trains with values in [0,1]∈R then provide a standardized waveform that we adopt as our data type, as shown in Figure 3c. This Figure shows the pipeline of data processing described above with sample waveforms for each step using the acceleration signal and focusing on the peak amplitude information.

The micro-movement spikes data type accounts for possible allometric effects given by the different anatomical features of the participants [14] and for minute fluctuations that grand-averaging methods smooth out as noise; so we can compare individualized movement-related variations across different participants without incurring in gross data loss.

### 2.4. Micro-Movements Spike Trains as a Gamma Process

**Figure 4:**
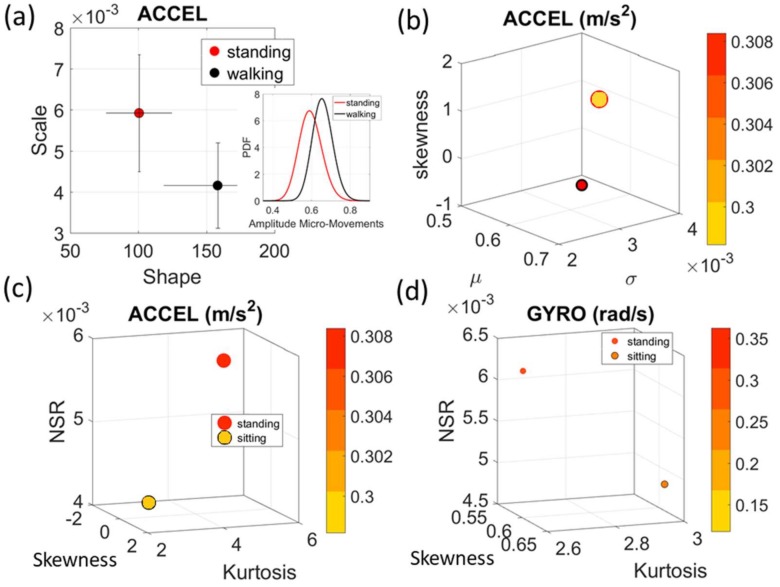
Micro-movements spike trains as a *Gamma* process. (**a**) The estimated *Gamma* shape and scale (*a*, *b*) parameters are plotted on the *Gamma* parameter plane with 95% confidence intervals for the Kaggle sample summary data (mean acceleration for standing and walking activities). Empirically estimated PDFs are insets. (**b**) The estimated *Gamma* moments (see text) with the color representing the range of median acceleration values in this example (m/s^2^) (**c**) Identification of parameter space separating the activities include the skewness, kurtosis and *Gamma* *b*-scale (NSR) for the acceleration peak amplitude (m/s^2^) and the angular velocity peak amplitude (rad/s). (**d**) Same as (c) for the gyro.

The normalized peak amplitude and peak timing fluctuations represented by real-valued spike trains ranging from 0 to 1, are treated as events in a continuous random process. They are studied under the general rubric of Poisson random processes. Specifically, we use them as input to a *Gamma* process and estimate the *Gamma* parameters. We then plot the estimated *Gamma* parameters with 95% confidence intervals on the *Gamma* parameter plane (Figure 4a). There we see, for example, that the noise to signa ratio (NSR) is higher for standing than walking and that the walking activity gives rise to more symmetric PDF, also shown in the inset with the corresponding PDFs. We also estimated the *Gamma* moments and plotted them on a space spanned by the *Gamma* mean along the *x*-axis; the *Gamma* variance along the *y*-axis; the skewness along the *z*-axis and represented in the marker size the value of the kurtosis. Since these estimations are performed using the normalized fluctuations in peak amplitudes and timings (independent of the sensor type or participant anatomical features) they span real values from 0 to 1. We retain the physical ranges (m/s^2^ or rad/s, or seconds) to color code the marker’s face and provide a color-coded gradient showing the range of parameter value (peak amplitude or peak timing) for each participant across each cohort. The color of the marker edge represents the median value of the parameter for activity type or task condition. Figure 4a–d shows the sample graphs for the data in Figure 3. Figure 4c,d identify higher moments of the data to build a parameter space further confirming the separation of the activities along the skewness–kurtosis–NSR dimensions. These types of plots will be used in the rest of the paper to provide instances of methods of parameter identification and data visualization.

## 3. Results

### 3.1. The Cohort of 30 Participants from Kaggle Activity Recognition Set

**Figure 5:**
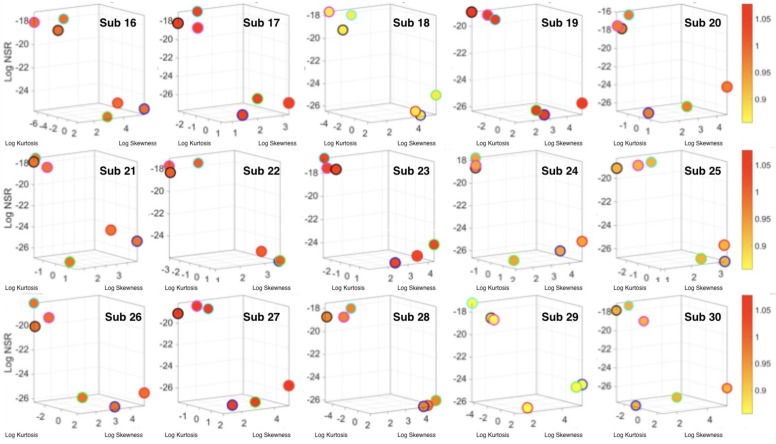
Individual results of the analyses of acceleration peak timings as micro-movements spike trains. Color represents median acceleration motion range across the cohort of 15/30 participants (the other 15 are in the Appendix B for better visualization). In each participant the timing stochastic signatures of standing, sitting and laying separate from those of walking, walking upstairs and walking downstairs.

**Figure 6:**
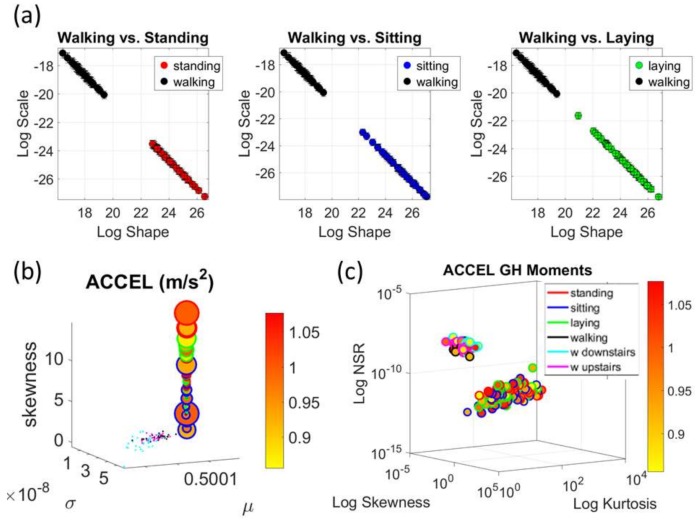
Comparison of tasks that require overt vs. covert movements. (**a**) Walking vs. standing, sitting or lying for all 30 participants plotted as points representing the distribution of spike trains of fluctuations in timing. (**b**) The corresponding *Gamma* moments estimated from the shape and scale parameters for each participant also separate and reveal differences in the skewness and kurtosis (marker edge color codes the task according to legend) in (**c**) where the *z*-axis represents the dispersion and separates the activities into two clearly self-emerging clusters of tasks with higher NSR (walking, walking upstairs and walking downstairs), whereas the skewness and kurtosis show a broader spread for tasks that require less overt motions (standing, sitting and laying).

The analyses of the data from the 30 participants revealed separation of activities involving less motion (standing, sitting, laying) from those involving more motions (walking, walking upstairs and walking downstairs.) This separation was evident in the micro-movement spikes derived from the fluctuations in the timing of the acceleration peaks and those from gyroscope angular speed. Figure 5 focuses on the timing data for the accelerometer for each participant. Figure 6a shows sample pairwise comparisons for these activities requiring fewer motions in relation to walking. Each dot represents a participant with tight 95% confidence intervals for the estimated *Gamma* parameters. Similar results were obtained for the timing of peaks in angular speed. The amplitude data showed some overlapping in the accelerometer case and more separation in the gyroscope case (not shown). Figure 6b shows the *Gamma* moments of all activities for all participants, with a clear separation expressed in the skewness and kurtosis (size of the marker) between the two classes of activities across the cohort. Figure 6c focuses on the separation along the NSR dimension using as additional dimensions the information that skewness and kurtosis provide. The activities involving walking, walking upstairs and walking downstairs, clearly clustered apart from those involving standing, sitting and laying. Note here that these clusters self-emerged from the stochastic signatures and the moments empirically derived from the variability inherent in the data. They were not obtained with ML clustering methods (e.g., K-means) or using predetermined training/testing sets and assumed similarity metrics to measure distance between neighboring points. Each marker represents a probability distribution function corresponding to the spikes train representing the inter-peak intervals of the participant’s acceleration peaks.

### 3.2. The Summary Mean Data from the Kaggle Activity Recognition Set

**Figure 7:**
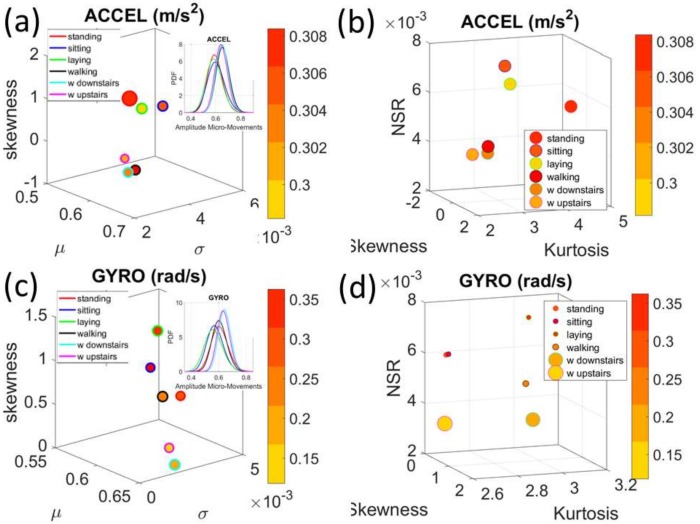
Comparison of acceleration and gyro signatures for the summary (group) data set involving the mean values of fluctuations in peak-amplitude. (**a**) Separation into two general types of activities (walking related and resting related) are congruent with the individual subjects’ data. (**b**) Separation is also evident for the higher *Gamma* moments. (**c**,**d**) Gyro data separates but does not show a clear cut pattern as the accelerometer data.

The data corresponding to the summary data using the mean acceleration in the Kaggle set also provided automatic classification of activities despite its coarser (averaged) nature. Figure 7a focuses on the data from the spike trains built from the fluctuations in amplitude. It provides two clusters congruent with the data from the 30 participants in Figure 5 and Figure 6 above. Here the activities also separate in the amplitude domain according to those tasks reflecting overt movements (walking type) and those reflecting covert (minute subtle) movements (standing, siting and laying.) The latter show higher levels of skewness and more variability than the former in the acceleration (Figure 7a). Figure 7b reflect this separation in the NSR levels whereby higher NSR levels are found in the activities of overt motions, with laying having the least amount of acceleration amplitude of the three in this cluster. The range of peak acceleration values is uniform across all three activities, as reflected by the size of the marker in Figure 7b.

In the gyro case reflecting fluctuations in amplitude of angular rotation, the activities also separate, but have no clear pattern as shown in the acceleration case. Figure 7c shows the differences in estimated *Gamma* moments using similar convention as above. Here the levels of angular rotation are highest during walking downstairs and lowest during laying, which also has the lowest skewness. Insets show the estimated PDFs. Further, the parameter space of skewness, kurtosis and NSR for the angular speed data produces the separation between the two movement classes reflected in these activities. The size of the marker echoes in this case the range of values (in rad/s) for each activity type. Larger rotational ranges in walking, walking upstairs and downstairs, contrast with those in the activities involving minute covert motions in Figure 7d.

**Figure 8:**
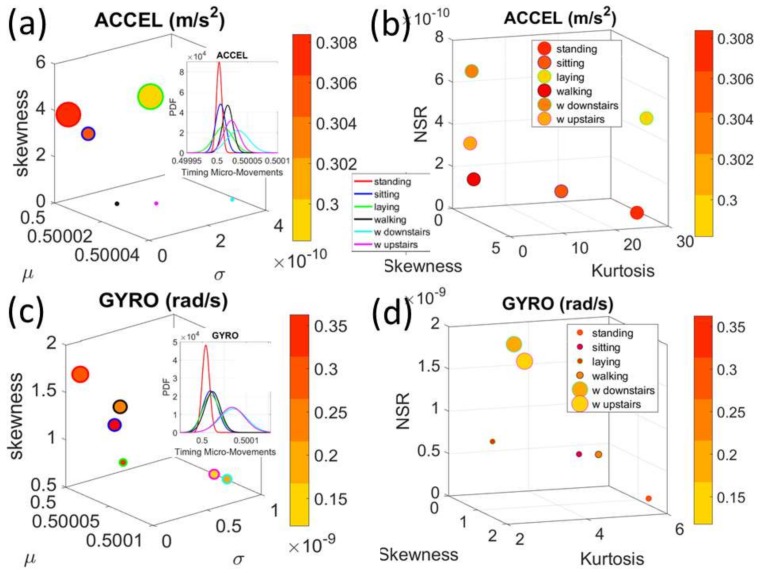
Comparison of acceleration and gyro signatures for the summary (group) data set involving the mean values of fluctuations in peak-timing. (**a**) Separation into two general types of activities (see legend and marker edge color). (**b**) *Gamma* skewness, kurtosis and NSR also distinguish across activities. (**c**,**d**) Gyro’s angular speed peak-timing related signatures distinguish between the overt and covert-movement related activities.

Figure 8 shows similar patterns for the timing domain. Here we show the same layout as in Figure 7 but focus on the fluctuations in the timings of the peaks.

### 3.3. The Data from Smart Shoes

**Figure 9:**
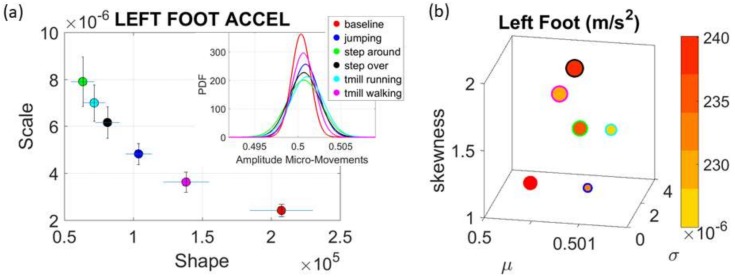
Comparison of different activities using Zeblok smart shoes. (**a**) *Gamma* parameters from left foot peak-timing spike trains by itself can distinguish walking normally (baseline) from walking on a treadmill and jumping. Other activities with more subtle requirements (walking around or over an obstacle) are also different from baseline walking. (**b**) *Gamma* moments further confirm these distinctions. Color gradient shows the range of deviations from the estimated mean (peak accelerations).

**Figure 10:**
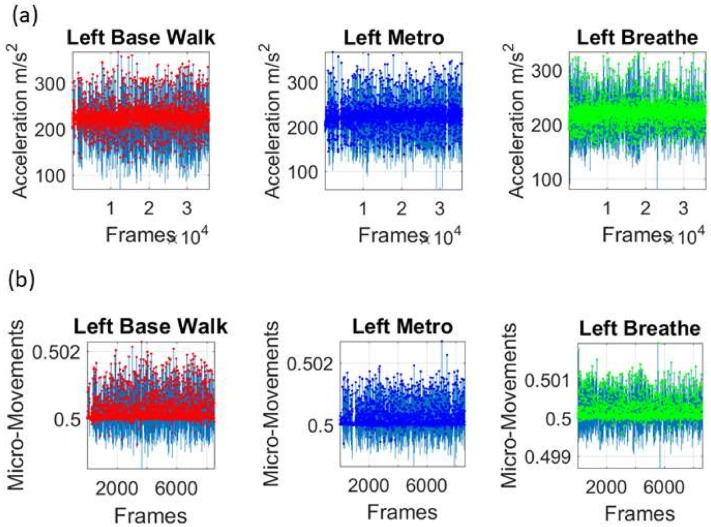
Comparison of subtle changes in walking patterns and levels of autonomous control using accelerometer data from the smart shoes Zeblok. (**a**) Raw data from each task (baseline walking: automatic; walking with a metronome in the background: spontaneous; walking with a metronome in the background while instructed to breathe to the metronome rhythms: deliberate). (**b**) Spike train micro-movements extracted from minute fluctuations in the peak timings.

**Figure 11:**
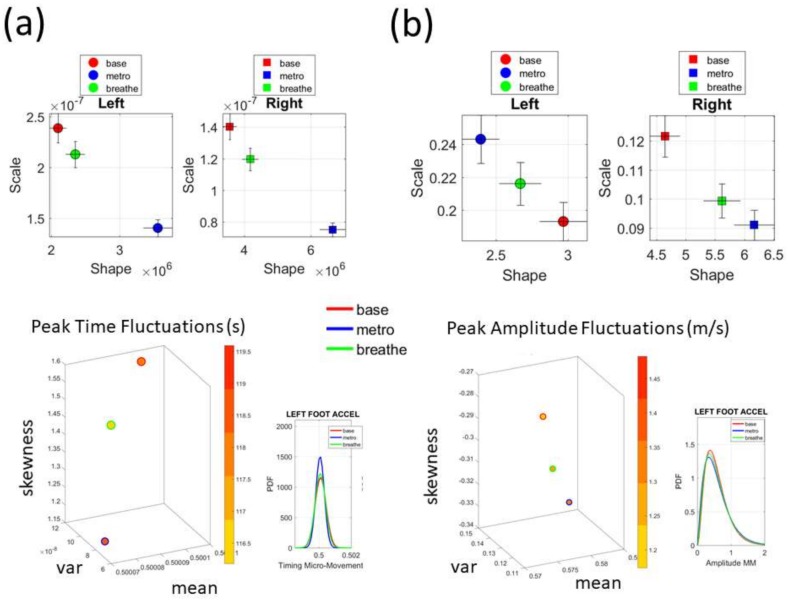
Comparison of subtle changes in walking patterns with task demands and levels of autonomous control using accelerometer data from the Zeblok smart shoes. (**a**) Stochastic signatures on the *Gamma* parameter plane and *Gamma* moments space for fluctuations in peak timing and (**b**) fluctuations in peak amplitude.

The accelerometer sensors in the insoles of the Zeblok shoes provided data to test the methods. Here a participant performed six different tasks and we measured the activity from each foot independently. Figure 9a shows the parameters on the *Gamma* parameter plane with 95% confidence intervals. Figure 9b shows the *Gamma* moments for the left foot. As before, the activities were separable with respect to the baseline walking according to the variability in peak timing, with visible changes even in the raw waveform (Figure 10). Congruent results were found for the fluctuations in peak amplitude. This is shown for both feet in Figure 11 (with panel 11(a) focusing on the fluctuations in peak timing and 11(b) focusing on peak amplitude.)

### 3.4. A Simple Task to Measure Levels of Control

Finally, we used the Zeblok shoes to test if they could pick up rather subtle differences in performance under different levels of autonomous control. The raw data and the spike trains for the fluctuations in peak timing are shown in Figure 10. In the first task (baseline walking) we assessed rather automatic motions and asked if they would spontaneously change in the presence of a metronome. While we did not instruct the participant or noted the presence of the metronome to the participant, our hypothesis was that the mere presence of this audible rhythm would entrain with the person’s biorhythms and shift the overall stochastic signatures. Likewise, we hypothesized that the deliberate condition, where the person was instructed to breathe to the rhythm of the metronome, would change the bodily biorhythms and this would likely be reflected on the feet patterns of linear acceleration.

To ascertain these possible effects on the variability of the data at this micro-level, we examined once again, the fluctuations in peak amplitude and peak timing. The latter consistently shifted the signatures of the left and right foot. This can be appreciated in Figure 11a on the estimated *Gamma* parameters. The former had different effects on the left and right foot Figure 11b. In both the timing and amplitude, we also examined the *Gamma* moments (plotted for the left foot on the bottom panels of Figure 11). We found marked differences across all parameters, with the metronome condition displaying the lowest variability and lowest NSR of all three versions. Remarkably, the insoles picked up these very subtle changes and systematically differentiated automatic from spontaneous from deliberate modes of control.

## 4. Discussion

The present work presented a new standardized data type (micro-movements spikes) and statistical platform for individualized behavioral analyses (SPIBA) in combination with data from Kaggle.com to illustrate the use of the methods. Further, to illustrate their general use with other commercially available sensors, we provided a task designed in our lab [13] to probe levels of autonomous control. This task examines the interplay between deliberate and spontaneous autonomy in relation to fully automatic behaviors under natural conditions. In this case, the general behavior is walking at one’s regular pace. In the spontaneous condition, the task probes self-emerging entrainment between the internally self-generated biorhythms and the external tempo of a metronome that the person is not instructed to follow. In the deliberate condition, the person is instructed to breathe at the pace set by the metronome. Both tasks evoked changes in the peak timings and the peak amplitudes such that their accumulation over the span of minutes, shifted the probability distribution function, best describing the process in an MLE sense. Using a *Gamma* process to characterize the micro-movement spike trains, these shifts were detectable. Their changes were tracked within the time span of minutes in each of the data sets we examined. The methods can provide a stochastic trajectory amenable to determine the rates of change of the signatures, a feature that is unique to the person’s nervous systems. In this sense, we have used these methods in the past to track a clinical trial in young children with SHANK3 deletion syndrome [15,16] and identified the pharmacodynamics of each participant to tailor the treatment and apply concepts from personalized medicine. Here we wanted to use the methods to examine other activities and ascertain whether the data type and analytics could automatically distinguish one task from another, even in cases where the differences may not be apparent.

The Kaggle data set provided us with two different types of tasks requiring fundamentally different motor control strategies. The tasks involving large, overt movements within the realm of walking allowed us to probe differences in the linear acceleration and angular speed domains owing to variations involving going up or down stairs in relation to natural walking. The methods picked up those subtle variations and systematically separated for each participant the three different versions of the walking task. The other set of tasks involving covert, small movements required keeping the body in a postural position (standing up against gravity, laying or sitting). These activities demanded a different type of motor control aimed at maintaining a posture, rather than dynamically changing it. As in the overt movements, using our methods, each participant data was automatically distinguishable by the activity type. Furthermore, for each participant, the two classes of tasks lied on different locations of the *Gamma* parameter plane and on extreme locations of the *Gamma* moments space. They could be unambiguously separated for each person and for the group. The summary data involving mean activity in another Kaggle version of this data set, also yielded automatic separation by activity type within each movement class. Several parameter spaces were presented where the variability inherently present in the data systematically separated the motor control features of these tasks.

**Figure A1:**
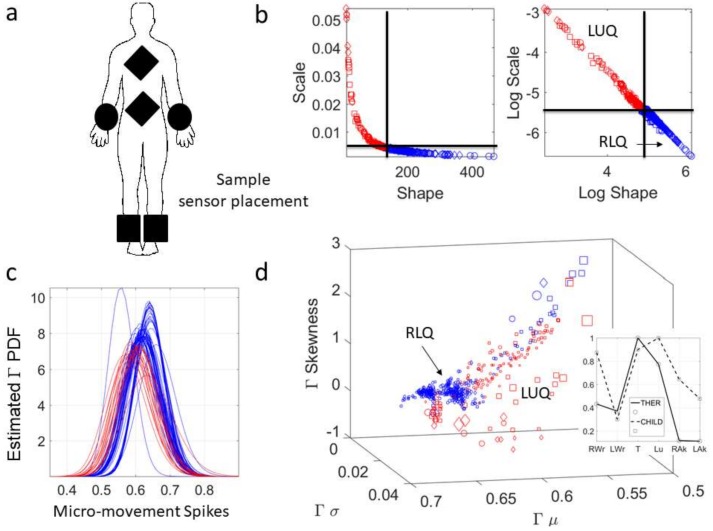
Criteria offered by the statistical platform for individualized behavioral analyses (SPIBA) and the micro-movements spike data type. (**a**) Generic sensor placement across the body (in this case wearable accelerometers were used synchronously and registering data for 60 min at 128 Hz APDM opals, Portland OR). (**b**) Estimated *Gamma* parameters and quadrants derived from the median values of the shape (*a*) and scale (*b*) dividing data into LUQ (high noise and towards the Exponential limiting range—random and noisy) vs. the RLQ (low noise and towards the Gaussian limiting range of the *Gamma* plane—predictive and low noise.) (**c**) Estimated PDFs for each case. (**d**) Note that the markers type denotes the sensor placement. The estimated *Gamma* moments also offer location of the probability landscape and within it the color-coded points identify the highly predictive data with low noise (blue) and distinguishes it from noisier and more random events. Inset obtains the ratio of RLQ to LUQ for two participants who interacted during a task, whereby the values below 1 denote prevalence of higher noise and more randomness in the patterns of the sensor type on the *x*-axis (Right Wrist RWr, Left Wrist LWr, Thorax T, Lumbar L, Right Ankle RAk, Left Ankle LAk.). In this case, we compare the two-people interacting in tandem and can tell which body part is most informative and predictive of future statistical events, vs. which body parts are more random and noisier.

**Figure A2:**
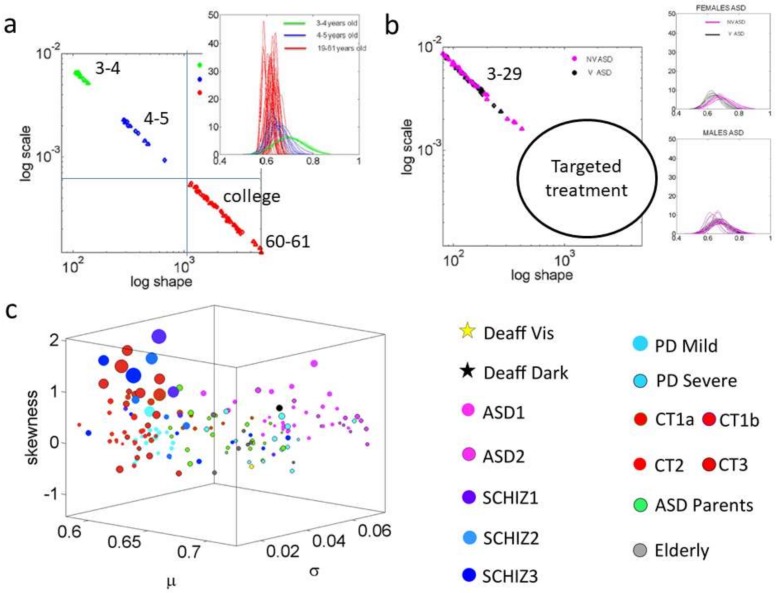
Taken from [3,7]. Mapping disorders of the nervous systems with the SPIBA framework and the micro-movement spikes derived from linear speed time series during continuous pointing behavior. (**a**) Normative cross-sectional data from 3 to 61 years of age demonstrates a maturation process. As age increases for the different participants in different age groups, the shape increases in value and the scale (noise to signal ratio) decreases in value according to a power relation well fit to the log–log scatter by a linear relation. (**b**) There is an absence of the neurotypical maturation process in the stochastic signatures of autism spectrum disorders or condition (ASD) pointing behavior. Regardless of age (3–29 years old), or verbal capacity (verbal and non-verbal), the ASD participants remain in the left upper quadrant (LUQ) with higher noise and more random features (nearing the Exponential distribution). (**c**) Taken from [3]. Localizing autism among other nervous systems pathologies.

**Figure A3:**
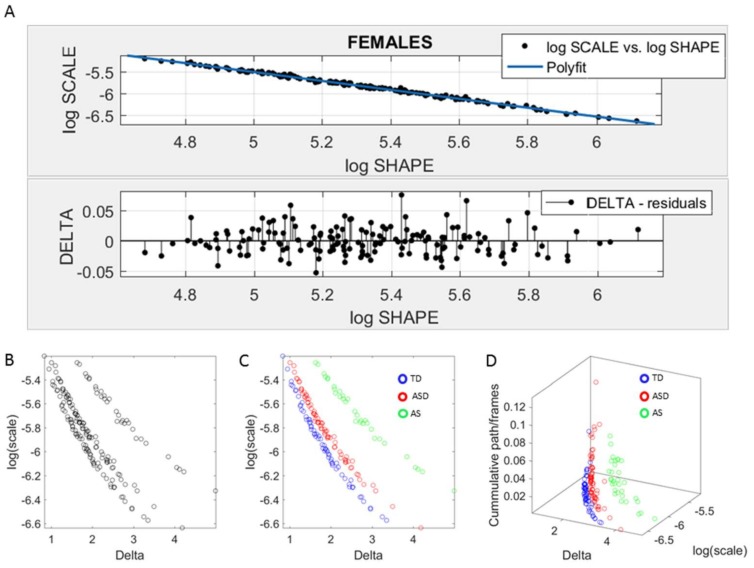
Taken from [24]. Characterizing the female phenotype with ASD and Asperger’s (AS) syndrome. (**a**) Line fit to the log–log *Gamma* parameter plane and delta-residuals. (**b**) Blind characterization of females according to delta residual from linear polynomial fit of the log–log scatter and the log of the estimated *Gamma* scale (noise to signal ratio). (**c**) The labels from the clinical data informing who is ASD or AS or typically developing (TD) separate the scatter according to the Diagnostics Statistical Manual, DSM-criteria. (**d**) Additional dimension on the *z*-axis denoting the cumulative path length of the head excursions across the full resting state time (300 frames at one hertz, i.e., five minutes trying to remain still but having too much involuntary head motions polluting their motor control.) Data from the Autism Brain Imaging Data Exchange (ABIDE I and II) repository (comprising over 2500 records with approximately one-fifth females).

We underscore that the clusters we found spontaneously self-emerged and were not required a priori (e.g., as with *K-means*.) Furthermore, we did not preset a testing and training set as ML methods do. Instead, we utilized the full data set from Kaggle without any pre-defined subsets or any additional features (e.g., the Kaggle set has 561 features and treats them as vectors, but it is not clear they are supported by a vector space, or provide a metric space, or that it is possible to extract similarity metrics, or what they should be, etc. The present approach provides a distribution-free type of analysis and rather empirically informs the stochastic model of inherent features present in the data variability (see also Appendix A Figure A1, Figure A2 and Figure A3). As such, the clustering of the activities self-emerges based on the types of neuro-motor control required by these tasks. In this sense, the results are congruent with the previously proposed notion of different movement classes in motor control that map well onto different levels of variability [5]. Movement variability is important in separating levels of autonomy that self-emerge during neonatal stages [6] and manifest throughout life, as the person acquires the type of volitional control [17] that is conducive of the development of the sense of agency [18,19].

In our previous research we had explored the present methods using smart phone data from a Kaggle set concerning patients with Parkinson’s disease and age- and sex-matched controls [20]. Here we extend the results to refine the classification of specific tasks. Within different activity types, we can further distinguish the statistical variations that subtle changes in a highly practiced motion such as walking, produce when performing it with deliberateness, vs. when evoking spontaneous entrainment with external biorhythms in the environment. In this sense, the task that we propose here can be used in combination with the tasks in the Kaggle set to provide a comprehensive profiling of the levels of autonomy of the nervous systems. As previously introduced by our group, these can go from entirely autonomic to entirely voluntary [5] and serve as markers of adequate neuro-motor control. These types of biometrics can complement clinical inventories like the Universal Parkinson’s Disease Rating Scale (the UPDRS) or the Autism Diagnosis Observational Schedule (ADOS) used to ascertain neurodegenerative or neurodevelopmental issues (respectively). Such inventories currently lack a component that assesses changes in data from waveforms self-generated by the nervous systems under consideration. In this sense we submit that nervous systems disorders at any stage of the human lifespan need to be physically measured. The types of biometrics this work offers are among those which would enable to track change in nervous systems activities along with detection of the shift in their stochastic signatures owing to treatments and/or disease progression. They can be considered as part of the ubiquitous computing (UBICOM) effort to connect research and technology to the service of unmet clinical needs.

Although the Kaggle set and the additional tasks that we introduced to test the methods and standardized data type take place in open loop settings, the promise of the present methods lies in the context of closed loop settings. In closed loop settings, the motor output activity is used as re-entrant input to influence the self-generated rhythms of the nervous systems. In such settings, we have parameterized the output, combined it with external sources such as audio-visual media and re-parameterized it as input to selectively steer various levels of autonomy in the system. These have ranged from spontaneous autonomy to deliberate autonomy [21], where we have been able to use the micro-movement spike trains and *Gamma* process to extract the preferred sensory input from the motor stream [22] and re-played it back in precisely re-parameterized ways, even beneath the person’s awareness. Utilizing these methods and data type have allowed us to explore each person’s capabilities for autonomous control. In turn, this heuristics-free approach to the motor control problem has provided us with new means to begin the path of defining objective indexes of autonomy and self-independence. These types of outcome measures are fundamental to help the person ascertain his/her sense of agency during activities of daily living, social encounters and life in general, as it dynamically unfolds from day to day. Future research using these new technological advances in the medical field will facilitate the development of objective criteria to define formal metrics of quality of life. Any treatment, clinical trial or basic science study will ultimately have to ascertain such criteria to help the person improve their existence and realize the full potential of his/her life.

### Potential Contribution to Behavioral Neuroscience

The analytics and data types introduced by this work may also be of use to researchers in behavioral neuroscience. Among other areas of basic scientific research, this field develops animal models for pre-clinical trials and at present lacks objective, heuristics-free methods to track the animal’s behaviors and to examine nervous systems activity at the periphery. Part of the potential benefit of adopting these analytics and data type is that they are derived with the cortical spikes in mind. As in the field of Computational Neuroscience focusing on (binary) cortical spikes trains, here we too define spikes from the behavioral kinematics data. In our case however, we provide a continuous real-valued way to describe the peak data and build a continuous scale between 0 and 1 to use continuous random processes for statistical inference. Our methods of objective and personalized behavioral analyses can thus be combined with the methods currently using continuous Poisson processes for the analyses of cortical spikes. In this way one could go beyond a “disembodied brain” approach to neuroscience and bring in this computational machinery adapted to the analyses of naturalistic continuous behaviors to redefine animal models for preclinical trials and animal research in general.

## 5. Conclusions

This paper provides a set of biometrics and standardized data types amenable for reproducibility and data exchange across labs and clinical settings that assess natural behaviors. Research programs that aim for a mobile health personalized concept to tailor treatments to the best capabilities and predispositions of the person’s nervous systems may also benefit from their use. However, most important of all, the methods offer new avenues to initiate the path of objectively defining indexes to help measure different levels of motor control when performing activities of daily life.

## 6. Patents

- Systems and methods for the diagnosis and treatment of neurological disorders. US PCT international application number 15/223,884 filed on 29 July 2016
- Objective and personalized longitudinal assessment of post severe traumatic brain injury. US patent application number 62/089,031 executed on 08 December 2014
- Systems and techniques for tracking neurodevelopment in infants. US provisional application number 62/408,542 filed 14 October 2016 (International PCT/US17/56779 filed 16 October 2017)
- Connecting peripheral and central nerves output signatures of variability through the same statistical platform. US patent application 62/409,943 filed 19 October 2016 (International PCT/US17/57365 field 19 October 2017).

## Figures and Tables

**Figure A4:**
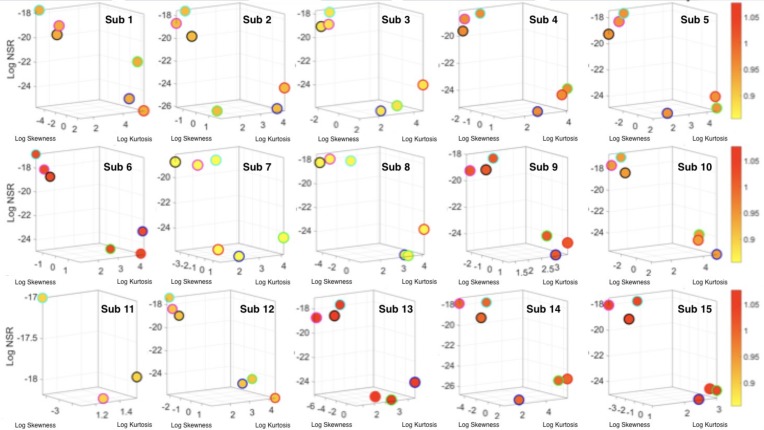
Remaining participants 15/30 from the full Kaggle.com cohort in Figure 5 of the main text.

## Appendix A

The *Gamma* parameter plane offers a new way to better understand the statistical inference for further interpretation of biophysical data. Since the *Gamma* scale (*b*) parameter is the noise to signal ratio, higher values of *b* indicate higher levels of noise to signal (estimated *Gamma* variance to estimated *Gamma* mean). Further, along the *Gamma* (*a*)-shape axis we can distinguish the limiting case to the left when *a* = 1 referring to the Exponential distribution, from the limiting case to the right referring to the Gaussian distribution. This enables us to distinguish events characterized by the most random distribution (the *Exponential*) whereby events in the past do not contribute to the prediction of future events, from cases where there is systematic prediction of future events from prior information. Figure A1a shows a possible way to place sensors across the body to map stochastic signatures of biophysical motion parameters. Figure A1b expresses the quadrant division of the *Gamma* parameter plane automatically determined by the median values of shape *a* and scale *b* as they shift with different sensory contexts on the *Gamma* parameter plane. Figure A1d shows the estimated *Gamma* moments and their corresponding regions color coded by quadrant subdivision. When a process remains in the left upper quadrant (LUQ) and very infrequently visits the right lower quadrant (RLQ) this provides empirical evidence that the process is primarily driven by spontaneous random noise. In contrast, when a process frequently transitions from the LUQ to the RLQ, this indicates a tendency towards the *Gaussian* range with lower noise to signal ratio and symmetric distributions.

This serves as a criterion to assess neurodevelopment (e.g., in autism spectrum disorders in Figure A2a) and identifies required targeted treatments. In other words, treatments that aim at shifting the signatures of the nervous systems towards the location in probability space where neurotypicals ended up (i.e., the maturation process that autistics did not undergo). Likewise, the map tells us about the location of such conditions in relation to other pathologies of the nervous system (expressed here in Figure A2b as the *Gamma* moments space map.) Lastly, we alert the reader of the power-law like relation empirically uncovered using these methods across thousands of participants (ages 5–60 years old) whereby as the shape of the distribution grows in value towards the Gaussian case, the dispersion (noise to signal ratio or scale parameter) decreases its value [23,24,25].

The slope of the fitting line to the log–log scatter conveys information useful to determine the rate of change of such power law relation. The delta-deviations from the linear fit to the log–log scatter also provide information about failure to follow this power relation between the shape and scale parameters. Figure A3 shows an example of such failure in females with ASD and Asperger’s diagnoses in relation to control females. This is important owing to the disparate ratio of five males to one female in the ASD subjective diagnosis. Here, involuntary head motions inside the scanner reveal a phenotypic feature of the Asperger’s and Autistic females as a departure from neurotypical age-matched female. Basic research has no information on the autistic female phenotype because of the 5:1 ratio [24].

Together the quadrants criteria involving the frequency of transitions and the magnitude of the shift, along with the slope criteria on rate of change and delta-deviation from the power relation, provide simple indexes to detect change and its rate across nervous systems’ self-generated biorhythmic activities along time-series data.
